# Enhancing Operating Room Skills and Confidence Among Third-Year Medical Students: A Quality Improvement Initiative

**DOI:** 10.7759/cureus.78189

**Published:** 2025-01-29

**Authors:** Matthew C Kane, Lillian S DeSousa, Deidre Pierce

**Affiliations:** 1 Surgery, East Tennessee State University Quillen College of Medicine, Johnson City, USA; 2 Internal Medicine, East Tennessee State University Quillen College of Medicine, Johnson City, USA

**Keywords:** anxiety, aseptic technique, confidence, education, medical student, operating room skills, sterility, surgery

## Abstract

Operating room (OR) sterility and proficiency are paramount in medical education, yet a standardized curriculum remains elusive. This study investigates the impact of an OR skills training workshop, led by students and supervised by OR staff, on third-year medical students' confidence and comfort in the OR setting. Third-year students at Quillen College of Medicine in Johnson City, Tennessee, participated in pre- and post-workshop surveys assessing comfort levels with key OR procedures. The workshop facilitated practice in a low-pressure environment, enhancing students' confidence significantly across six fundamental OR skills (p < 0.05). Strengths of the workshop included facility tours, hands-on practice, and mentorship by OR staff, while recommendations focused on smaller group sizes and additional skill coverage. Ongoing data collection aims to assess the workshop's long-term impact on clerkship experiences. This study underscores the importance of structured curriculum enhancements in surgical medical education.

## Introduction

Research suggests a high prevalence of depression and anxiety among North American medical students, particularly in those exposed to acute care surgery services. This exposure often leads to heightened levels of anxiety, which can adversely affect their perception of surgery as a potential career path [1,2]. In an exploratory survey conducted by Hill et al., medical students reported associating surgery with competition, stress, and significant personal sacrifice [3]. These perceptions and experiences can have a profound negative impact on their willingness to pursue surgery as a career.

It is estimated that 50% of medical schools are failing to meet the goals of teaching and evaluating technical procedures [4]. The structure and administration of surgical education curricula vary significantly across the United States, resulting in most medical students lacking a formal introduction to surgical skills and techniques [5]. Furthermore, students' procedural experiences vary greatly [6], with over 75% of medical students in two separate studies reporting insufficient training in basic surgical skills during their education [7,8]. The focus on fundamental surgical and procedural training is often diminished by a preclinical curriculum that prioritizes generalized skills and knowledge, such as conducting thorough histories and performing accurate physical exams [8,9].

At East Tennessee State University (ETSU) Quillen College of Medicine, we created a student-led OR skills class designed to enhance the surgical competencies of third-year medical students before they commence their surgery clerkship. By equipping students with essential operating room (OR) skills in a low-stress environment, we aim to improve their performance, confidence, and overall educational experience during their clerkship. Learning basic OR skills in a low-stress, supportive environment may significantly reduce the intimidation factor associated with the OR. This preparatory training allows students to practice and master fundamental techniques without the pressure of real-time clinical demands. By doing so, students can build confidence and competence before entering the actual OR, thereby enhancing their readiness and reducing anxiety.

## Materials and methods

IRB approval

Institutional review board (IRB) approval was obtained through ETSU IRB with approval number 0323.4E.

Workshop design

During the first week of the surgery clerkship, third-year medical students were invited to participate in an OR skills training workshop. The workshop was designed to provide hands-on practice in a low-pressure environment, using an actual OR room to simulate real-life conditions. The skills covered included patient transfer and positioning, performing a surgical scrub, donning a sterile gown and gloves, using aseptic technique, and holding retractors. The workshop also included a tour of the facility and teaching points from OR staff about the environment and culture of surgery.

As students arrived at the facility they were greeted at the front entrance by one of our student representatives. Students were handed a folder containing a consent form, a pre-workshop survey (see Appendix A), and a post-workshop survey (see Appendix B), each with matching identifying numbers at the top. The students were instructed to fill out the pre-workshop survey and consent form and place them face down in a closed drop box. Students were instructed not to put their names or any identifying information on the survey forms. Students kept the post-workshop study to be filled out after completion of the workshop. They were then guided through the hospital to the surgery department. Students were shown where the locker rooms were and instructed to change into surgical scrubs. 

Students were then shown where to find proper PPE such as scrub caps, masks, shoe covers, and eye protection. They were taken on a tour through the surgery department and shown the location of the charge desk, sub-sterile areas, storage areas, and operating rooms. Once oriented, students were taken to an operating room that was not currently in use and divided into two groups. One group would be taken to the scrub sinks and would practice a five-minute surgical scrub, while the other group went into the OR where a few surgical techs and OR nurses would walk them through the process of gowning and gloving and teach them about sterile technique and OR etiquette. After each student had performed these tasks, the groups switched places, and the teaching was repeated. 

Upon completion of both portions of the workshop, students were asked to complete the post-workshop survey and place it again face down in the closed drop box. Notably, this workshop took place in the surgery department that the students would be working in, but without a patient or any faculty present, for the purpose of creating a low-stress environment to practice these basic OR skills. 

Following completion of the surgery clerkship, all students, including those who did not participate in the workshop, were sent a post-clerkship survey via email to be completed via RedCap. These results were not paired with their previous surveys but were used to compare the clerkship experience of those who did and did not complete the workshop and to provide free responses that could help improve the workshop moving forward. 

Survey methodology

Students' comfort levels with these procedures were assessed using pre- and post-workshop surveys. The surveys utilized a 1-10 Likert scale, with 1 indicating minimal comfort and 10 indicating high confidence. In addition, post-clerkship surveys were administered to both workshop participants and non-participants to evaluate the workshop's long-term impact on their clerkship experience. Pre- and post-workshop surveys assessed confidence in patient transfer and positioning, performing surgical hand scrub, donning gown and gloves, using aseptic technique, holding retractors, and participating in the OR, while the post-clerkship survey added free-response questions regarding adverse experiences on the surgery clerkship. In total, 53 participants completed the workshop, with all 53 completing the pre-workshop survey and 27 completing the post-workshop survey. There were 48 responses to the post-clerkship survey, including 35 who participated in the workshop and 13 who did not participate.

Data analyses

Wilcoxon signed-rank test analyses were performed comparing student comfort level with the various technical skills between the pre- and post-surveys. A significance level of 0.05 was set for all hypothesis tests. All data analysis and manipulation were performed using Microsoft Excel (Microsoft Corporation, Redmond, WA, USA). 

## Results

Quantitative findings

The response rate of the study was approximately 50%. All respondents completed the pre-workshop survey (n = 53); however, not all respondents (n = 27) completed the post-workshop survey. There were 48 responses for the post-clerkship survey, including 35 who participated in the workshop and 13 who did not participate. The post-clerkship data were not included in analyses and were used primarily for feedback to improve the workshop curriculum. Wilcoxon signed-rank analysis of 27 participants who completed both the pre- and post-workshop surveys revealed a significant improvement in the students' comfort levels across four fundamental OR skills following the workshop (p < 0.05, z < -1.96 or >+1.96). Before the workshop, the median comfort levels ranged from 3 to 5. Post-workshop, these levels increased to a range of 5 to 9, indicating a substantial boost in confidence. Wilcoxon signed-rank test was used to analyze the absolute change in the median by calculating the difference in the post-test and pre-test. If a participant's score had a change of zero, their data point was discarded as it would not add any additional data to the result but skew to the total participant value. The difference in median change was determined to be significant in all five fundamental OR skills as displayed in Table 1. Thus, the workshop intervention had a statistically significant effect on Likert scale responses. 

**Table 1:** Wilcoxon signed-rank test results The comfort level for each skill was assessed on an entrance and exit survey using a 1–10 Likert scale, with 1 indicating no comfort and 10 indicating confidence (n = 27). Wilcoxon signed-rank test was used to analyze data comparing pre- and post-survey results at a significance level of <0.05. Subjects with the same test score in both the pre- and post-surveys were discarded. Z-score and p-value displayed above indicate that all results are significant, and the critical z-value at the 95% confidence interval is z = 1.96. *p-value significant at <0.05.

Skill	Post/pre-test		n	Mean rank (SD)	Rank sum	z	p*
Patient transfer and positioning	Negative rank		5	11.3 (6.82)	56.5	-2.05	0.04036
-	Positive rank		16	10.9 (6.16)	174.5	-	
	Total		21	-			
Surgical hand scrub	Negative rank		1	2.0 (0)	2	-4.31	< .0001
-	Positive rank		24	13.4 (7.05)	323	-	
	Total		25	-			
Gowning and gloving	Negative rank		3	5 (3.46)	15	-4.07	< .0001
-	Positive rank		23	14.6 (7.26)	336	-	
	Total		26	-			
Aseptic technique	Negative rank		2	4.7 (3.18)	9.5	-4.21	< .0001
-	Positive rank		24	14.2 (7.33)	341.5	-	
	Total		26	-			
Holding retractors properly	Negative rank		5	14.3 (5.92)	71.5	-2.44	0.014
-	Positive rank		20	12.6 (7.62)	253.5	-	
	Total		25	-			
Participating in the OR	Negative rank		5	7.0 (3.96)	35	-3.69	0.0002
-	Positive rank		22	15.5 (7.68)	343	-	
	Total		27	-			

Qualitative feedback

Free response feedback highlighted several strengths of the workshop, including *facility tours*, providing students with a guided tour of the OR facilities; *hands-on practice*s, allowing students to practice skills in a stress-free environment; *mentorship*, having OR staff present to teach and mentor students; and *proximity to live surgery*, conducting the workshop close to the time students would participate in live surgeries. Students also provided recommendations for improvement, such as *smaller group sizes,* to enhance individual attention and learning; *pre-workshop information*, providing preparatory materials before the workshop; and *additional skills coverage*, including more skills like suturing and providing information about surgical instrumentation.

## Discussion

The primary objective of our student-led OR skills class was to mitigate the intimidating nature of the OR by equipping third-year medical students with fundamental surgical skills in a low-stress environment. Our findings indicate that this initiative significantly enhanced students' confidence and preparedness for their upcoming surgery clerkship. By providing a space where students could practice and refine their skills without the pressure of being in a real OR, we aimed to reduce anxiety and build a solid foundation of competence.

Analysis of existing literature

It has been demonstrated that procedural performance and independence contribute significantly to a sense of competency in medical students [9]. A strong correlation exists between the frequency of task performance and self-assessed competence, suggesting that repeated practice is essential for building confidence and skill proficiency [10]. Therefore, it is paramount to create opportunities for medical students to practice and walk through various procedures in a controlled environment, ensuring they achieve a level of comfort and confidence before entering more high-pressure settings.

Surgical skills workshops, particularly those organized by students and led by experienced faculty, have shown promising results in boosting interest in surgical careers among medical students who were initially undecided about pursuing surgery [11]. Such workshops not only provide hands-on experience but also foster a supportive learning environment where students can ask questions and receive immediate feedback. In addition, Bowery et al. demonstrated that for learning in the OR to be effective, it is necessary to address factors that contribute to negative emotions, such as unfamiliarity with the environment, concerns about violating protocols, and organizational issues [12]. These factors can significantly impact a student's learning experience and overall performance in the OR.

Our findings align with existing literature that emphasizes the importance of deliberate practice and continuous feedback in surgical education. Ericsson's theory of deliberate practice highlights the necessity of focused, repetitive practice with continuous feedback to achieve expert performance [13]. Similarly, Stefanidis and colleagues have shown that proficiency-based laparoscopic simulator training, which involves repeated practice until a specific level of competence is achieved, leads to better skill retention and transferability [14]. These principles underscore the need for multiple, iterative training sessions in our workshop.

Simulation-based education has been widely recognized as an effective method for improving surgical skills and confidence [15]. Seymour et al. demonstrated that virtual reality training significantly improves operating room performance [16]. Kneebone et al. also emphasized the value of integrating simulation into medical education to bridge the gap between theoretical knowledge and practical skills [17]. By incorporating advanced simulation techniques and providing continuous feedback, we can enhance the effectiveness of our workshop and better prepare students for real-world surgical experiences.

Quantitative findings

Our study demonstrated a significant improvement in students' comfort levels across multiple fundamental OR skills following the workshop. With a response rate of approximately 50%, all respondents (n = 53) completed the pre-workshop survey, while a subset (n = 27) completed the post-workshop survey. Wilcoxon signed-rank analysis of these 27 participants revealed a statistically significant increase in comfort levels for all assessed skills, with p-values less than 0.05 (z <-1.96 or >+1.96). The median comfort levels increased notably from a range of three to five pre-workshops to five to nine post-workshops, underscoring a substantial boost in confidence.

Despite these encouraging results, our study had several limitations. The voluntary nature of participation in both the workshop and the survey may have introduced selection bias. The response rate for the pre- and post-surveys was approximately 50%, and the participants who completed both surveys might have been more inclined toward surgery and more experienced at baseline. This could have influenced their comfort levels and the overall outcomes. In addition, the subjective nature of self-reported comfort levels poses a challenge in drawing definitive conclusions. Self-assessment can be influenced by individual confidence levels and personal perceptions of competence, which may not accurately reflect actual skill proficiency.

Another limitation is the constraint of single-session training for OR preparedness. Research indicates that one-time training sessions often fail to produce consistent, long-term proficiency in surgical skills [18]. Mastery of surgical skills requires repeated practice and reinforcement over time. Future iterations and potential integration of this workshop into the medical school curriculum should incorporate multiple sessions prior to the start of clerkships. This approach would allow for progressive skill development, continuous feedback, and better retention of learned techniques.

Qualitative findings

The qualitative feedback from the participants highlighted several strengths of the workshop, including guided tours of the OR facilities, hands-on practice in a stress-free environment, mentorship from OR staff, and the timing of the workshop close to live surgeries. These elements contributed to creating a supportive learning environment that facilitated skill acquisition and confidence building. Mentorship has been shown to be a critical component of effective surgical training [19]. The presence of experienced OR staff to teach and mentor students provided invaluable real-world insights and guidance.

Participants also provided valuable recommendations for improvement. They suggested smaller group sizes to enhance individual attention and learning, provision of preparatory materials before the workshop, and inclusion of additional skills such as suturing and information about surgical instrumentation. These suggestions align with existing literature, which emphasizes the importance of tailored, repetitive training, and comprehensive skill coverage in surgical education [13,14]. Smaller group sizes can facilitate more personalized instruction and immediate feedback, which are crucial for effective learning.

Future directions

To address the limitations identified and further enhance the workshop's effectiveness, several strategies can be implemented. Research on deliberate practice and proficiency-based training suggests that incorporating multiple training sessions could lead to better skill retention and transferability [13,14]. In addition, expanding the workshop to cover a broader range of surgical procedures and scenarios, as recommended by participants, could better prepare students for the diverse situations they will encounter in the OR.

Moreover, integrating advanced simulation techniques and providing continuous feedback can bridge the gap between theoretical knowledge and practical skills. This ensures that medical students are better prepared for the rigors of surgical clerkships and, ultimately, their future careers in surgery. By incorporating these elements into our workshop, we can create a comprehensive, effective training program that meets the needs of medical students.

As this is an ongoing study, future research will focus on three main factors, namely, longitudinal impact, curriculum development, and broader implementation. Longitudinal impact will be assessed by examining the long-term benefits of the workshop on students' clerkship performance and interest in surgical careers. Curriculum development will involve refining the workshop based on feedback and potentially developing a standardized OR skills curriculum. Finally, we will evaluate the feasibility of implementing similar workshops across other medical schools to standardize OR training.

In conclusion, while our student-led OR skills class has shown positive outcomes in enhancing student confidence and preparedness, there is room for improvement. By addressing the identified limitations and expanding the workshop's scope, we can ensure that medical students receive comprehensive, effective training. Implementing multiple training sessions, incorporating advanced simulation techniques, and expanding skill coverage will be crucial for the future success of this initiative.

## Conclusions

The OR skills training workshop significantly improved third-year medical students' confidence and comfort in performing basic OR skills (p < 0.05). While the immediate benefits are clear, ongoing data collection over the next year will help determine the long-term impact of the workshop on students' clerkship experiences. This study highlights the need for continued curriculum enhancements in surgical medical education to better prepare students for their roles in the OR. This quality improvement initiative aims to demonstrate the value of structured, hands-on OR training in medical education, ultimately contributing to better-prepared, more confident future surgeons.

## Figures and Tables

**Figure 1:**
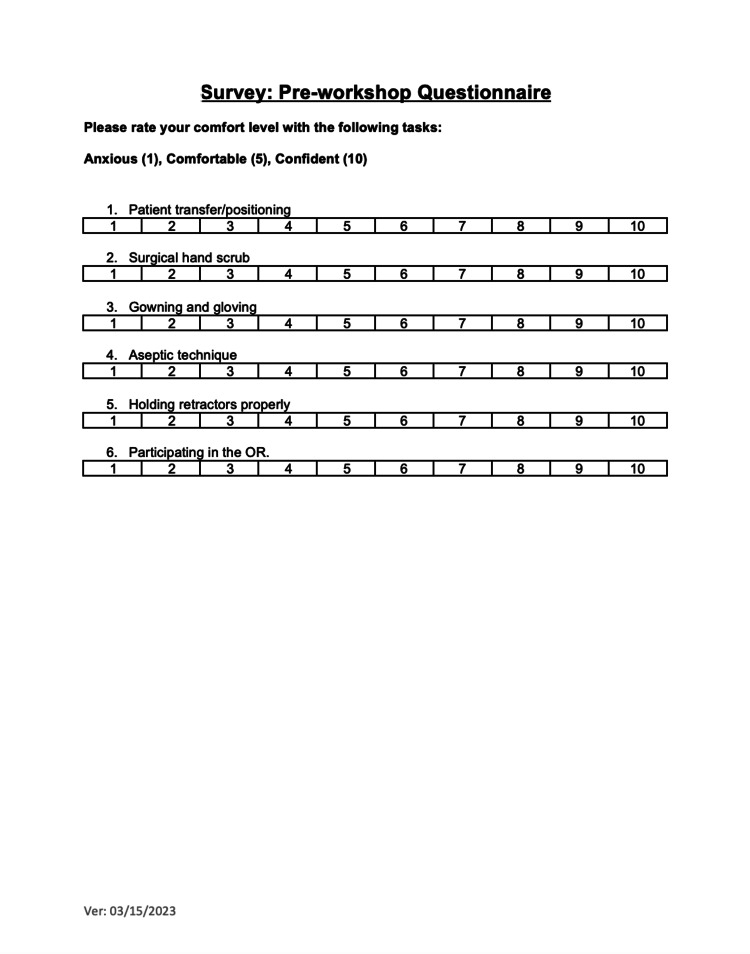
Pre-workshop questionnaire

**Figure 2:**
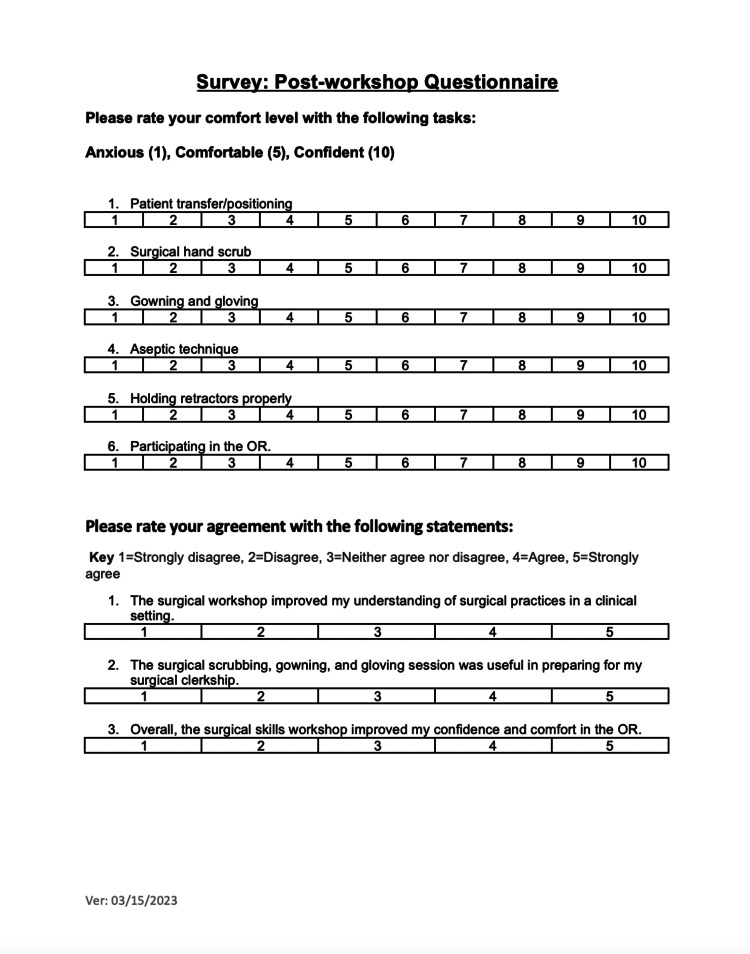
Post-workshop questionnaire (part 1)

**Figure 3:**
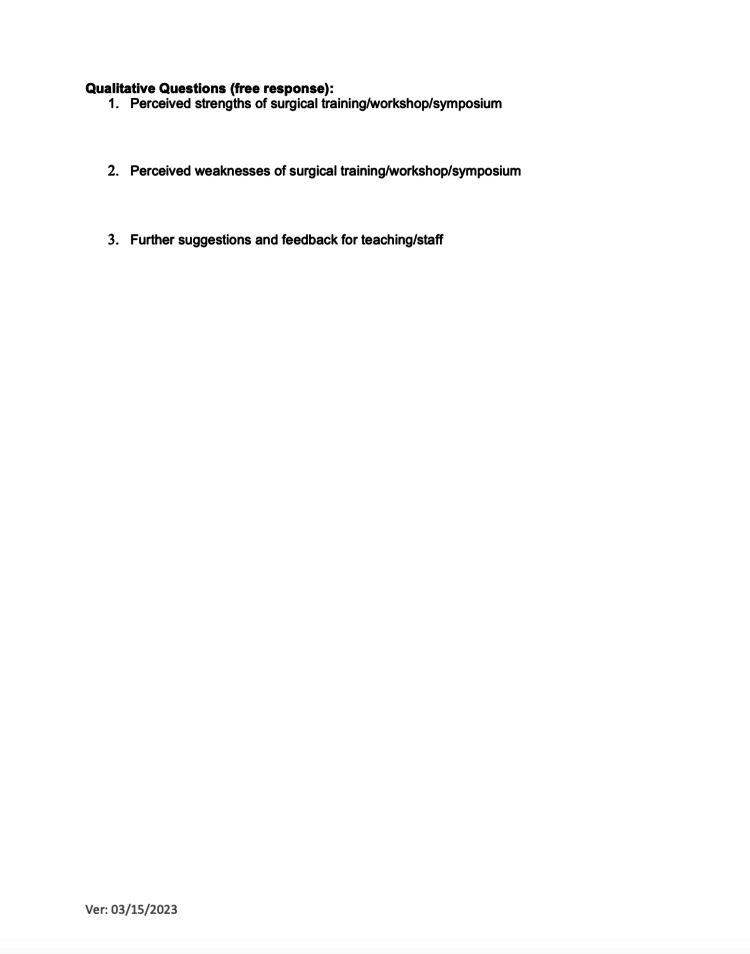
Post-workshop questionnaire (part 2)

## Appendices

Appendix A

Appendix B
